# Manure odor profiling for flock-level monitoring on commercial layer pullet farms: Vaccination events as a model stressor

**DOI:** 10.1016/j.psj.2024.104681

**Published:** 2024-12-16

**Authors:** Lara A. van Veen, Henry van den Brand, Anna C.M. van den Oever, Bas Kemp, Mara Meisenburg

**Affiliations:** a Vencomatic Group, Meerheide 200, 5521 DW Eersel, The Netherlands; b Adaptation Physiology Group, Wageningen University & Research, P.O. Box 338, 6700 AH Wageningen, The Netherlands

**Keywords:** Rearing hen, Manure volatile, Intestinal health, Precision livestock farming, Vaccination challenge

## Abstract

Continuous, non-invasive, and objective methods to detect flock-level responses to stressors, including intestinal health deviations, are currently lacking in poultry. This proof of principle study investigated the potential of manure odor profiling in monitoring stress responses in Lohmann Brown Classic, Lohmann LSL Classic and Lohmann LSL Lite layer pullets. Stressors were represented by a *Salmonella* vaccination given to the pullets at wk 3 of age (Dataset 1 and Dataset 2) and wk 16 of age (Dataset 4) and a viral/bacterial vaccine cocktail given at wk 12 of age (Dataset 3). Fresh manure was sampled daily, from 2 days before vaccination to 4 days after vaccination, with 4 pooled manure samples per day per dataset. Manure volatiles were concentrated into stainless steel sorbent tubes and analyzed by a thermal desorption system coupled with gas chromatography-mass spectrometry. Dataset, breed and sample location did not affect the manure volatile profiles before vaccination. Age did affect the manure volatile profiles, with beta-camphor, (Z)-6-Tridecene and serinol peak intensities elevated in young pullets and carbonyl sulfide and trimethylamine elevated in older pullets. *Salmonella* vaccination at wk 3 of age led to the most consistent changes in volatile peak intensities. (Z)-6-Tridecene was decreased in Dataset 1, Dataset 3, and Dataset 4 after either the *Salmonella* vaccination or the viral/bacterial vaccine cocktail, despite differences in hen age and house. The injected viral/bacterial vaccine cocktail created a more clear time-dependent shift in the manure volatile profile than the orally-provided *Salmonella* vaccination in older pullets at resp. wk 12 and 16 of age. No overlapping trends in upregulated or downregulated volatiles were found between all datasets. To conclude, volatile profiles of rearing hen manure are affected by vaccinations as a proxy for stressors, and the magnitude and direction of the response depends on the age of the pullets at vaccination, the vaccination method, and the pathogenic properties of the vaccine. The reduced peak intensities of volatiles after vaccination in 3 out of 4 flocks suggests the potential of manure odor profiling in monitoring stress responses in layer pullets.

## Introduction

In recent years, there has been a growing interest in understanding and improving intestinal health in poultry. New challenges for intestinal health have emerged with the housing of laying hens in cage-free systems, including the increased transmission of bacteria, viruses and parasites with high infection loads via the litter, air and aviary system (Bonnefous et al., 2022; Zhao et al., 2016). Maintaining optimal intestinal health is crucial for the general health and welfare of laying hens and for the farm economics. Laying hens have been selected for optimal feed conversion and economic consequences of suboptimal feed use are high, especially with extended laying cycles (Traore and Doyon, 2023). Subclinical intestinal infections -for example, those caused by endoparasites- can result in economic losses (Shini and Bryden, 2021) due to inefficient nutrient utilization (Das et al., 2010) and secondary infection with *Salmonella* (Chadfield et al., 2001). *Eimeria* infections can temporarily cease the egg production of laying hens (Sharma et al., 2024). Conversely, optimal intestinal health increases resilience against stressors and might reduce for example the risk of injurious feather pecking development, a major welfare concern that increases with the use of non-beak-trimmed hens (Mens et al., 2020; van der Eijk et al., 2020). To prevent negative impacts on intestinal health, housing high-productive hens in cage-free systems requires optimized management, such as increased control over feeding regimes and climate regulation (Ricke et al., 2022; Tierzucht, 2023). A tight vaccination scheme is used to protect (intestinal) health of hens (Kaufmann-Bart and Hoop, 2009). Although vaccinations are critical for the long-term protection of hens against pathogens, their effectiveness depends on proper administration and management.

Despite signals from the field about the health and welfare impacts of vaccinations in pullets, few studies have focused on vaccination as a stressor. *Salmonella* vaccination seems to affect cecal microbiome composition (Lyimu et al., 2023), but is not known to induce clinical symptoms in layers. In contrast, Lee et al. (2005) showed that subcutaneous vaccination of 2-week old layer pullets with *Staphylococcus gallinarum* resulted in a lower body weight at wk 5 of age compared to unvaccinated pullets. Several vaccinations require handling of the hens, which increases stress levels with consequences for intestinal health via the gut-brain axis (Beldowska et al., 2023). Vaccinations can therefore be regarded as a stressor that has both direct and indirect impacts on the intestinal health in poultry.

Given the impact of environmental and management-related stressors on poultry, there is a need for reliable assessment of intestinal health of laying hens to maximize health, welfare and productivity. Intestinal health assessment can include invasive and non-invasive methods. Invasive methods of intestinal health analysis require culling of hens and tissue sampling for biomarker assessment (Ducatelle et al., 2018). Non-invasive analysis of manure samples for the presence of *Salmonella* is regulated via European-Union coordinated programs (EFSA), especially because of the implications of *Salmonella* infections for human health. The majority of laying hen farmers, veterinarians and other poultry experts use visual observations of manure and litter quality as indicators of laying hen intestinal health (van Veen et al., 2023). In the latter study, some farmers indicated that they additionally use olfactory cues from the laying hen house for health and welfare assessment, e.g. to detect *Escherichia coli* infections based on the manure odor profiles.

Manure odor profiling could pose an opportunity for advancements in poultry health assessment. For example, volatile organic compounds released from the manure can reflect the intestinal microbiota composition (Bernard et al., 2024), (patho)physiological metabolic processes of the host (Kim et al., 2020), and the interaction between host and microbiota (De Lacy Costello et al., 2008). Studies have characterized volatile profiles of broiler manure and broiler farm litter (Beauclercq et al., 2019; Garner et al., 2008; Sharma et al., 2018; Yasuhara, 1987) and air (Murphy et al., 2014; Trabue et al., 2010). No studies exist that characterized the complete volatile profile of laying hen manure in vivo. Despite the lack of studies examining the volatile profile of laying hen manure, research on broilers provides valuable insights into how health status can affect the manure volatile profile. In broilers, several studies have demonstrated that various diseases and health conditions can significantly alter the volatile profile of manure and litter, for example in the case of gastro-intestinal disturbance by *Campylobacter* (Garner et al., 2008) and *Necrotic enteritis* (Sharma et al., 2018). Bombail et al. (2018) found that rats can detect stress-induced odors in broiler feces after subjecting them to negative postnatal experiences, suggesting that general stress responses of chickens can affect manure odor profiles. These findings together highlight that manure volatile profiling could be a promising non-invasive tool for monitoring poultry gut health and deviations in health, underscoring the need for further exploration in laying hens.

Continuous, non-invasive, and objective methods to detect changes in intestinal health on the flock-level are currently lacking in poultry. One technology that could facilitate non-invasive monitoring is the electronic nose (**e-nose**) system. This technology mimics the olfactory system, measuring and identifying specific odor profiles in air, the manure, or other substrates. E-nose systems have been utilized to detect *Salmonella enterica* in broiler manure in a laboratory setting after artificial infection of manure samples (Kizil et al., 2015). Sohn et al. (2008) showed that an e-nose could monitor air odor semi-continuously in a broiler house, but highlighted that external factors, such as season, housing design and litter type affect volatile profiles and volatile concentrations, which hampers its implementation in practice. Grilli et al. (2018) developed a prototype e-nose to detect *Eimeria* infection based on air in broiler houses at an early stage. However, the air in the poultry house is a complex mixture of compounds with temporal and spatial concentration fluctuations (Dunlop, 2011; Dunlop et al., 2016; Trabue et al., 2010), which are not all related specifically to chicken health status. This complexity highlights the need for more targeted approaches that can provide specific health indicators for laying hens in large groups.

To address these challenges, focusing on direct sources of health-related volatiles, such as volatiles released from manure, may offer a more precise and reliable assessment of poultry health. Development and integration of an on-site e-nose for laying hen health assessment requires multiple research phases. This study is a first step to assess the potential of manure volatile profile as a continuous, reliable indicator for monitoring hen intestinal health in commercial non-cage laying hen systems. As a proof of principle, several vaccinations were considered as events that could cause deviation in hen health during rearing, either directly by affecting intestinal health or indirectly by causing handling stress to the hens.

The primary objective of this proof of principle study was to investigate relationships between management-related stressors, induced by 2 types of vaccinations at 3 different ages, and the volatile profile of pullet manure on flock-level. To address this objective, it was first assessed whether or not regular manure volatile profiles are comparable between different layer breeds, sample locations in the rearing hen house and the age of the pullets. Secondly, it was studied whether or not manure volatile profiles change due to vaccination at different ages, and in which direction. Thirdly, volatiles were identified that showed a change in peak intensities after vaccination. Lastly, it was studied whether or not the direction of change in peak intensities was consistent across ages and vaccination events, and what these changes might imply for laying hen health. Consistent changes in shared volatiles could indicate similar responses of pullets to stressful events.

## Materials and methods

### Study design

Three commercial rearing hen houses (House 1, House 2, House 3), located on 2 farms in the Netherlands, were used for this study. Layer pullets were reared from day 0 (arrival at rearing hen farm) in aviary-type systems that consisted of 3 tiers with a manure belt at each tier. In the first weeks of age, the pullets were locked up in the system per tier, whereas access to a litter area was provided from wk 4 onwards. The number of pullets housed ranged between 25,000 and 75,000 pullets per house (Table 1). Different breeds of pullets were used in this study, with House 1 and 3 containing Lohmann Brown Classic hens in all rows, and House 2 containing Lohmann LSL Classic in 2 rows, Lohmann LSL Lite in 1 row and Lohmann Brown Classic in 1 row. The study took place between November 2022 and April 2023. All flocks followed the normal management and feeding procedures during the study according to guidelines from breeding companies and feed producers. Houses 1, 2, and 3 employed manure belt aeration systems, whereas House 4 did not. Outdoor fresh air entered all houses through inlets at the side, and exited via ventilators positioned at the backside of the house. The ventilators were regulated to maintain an optimal house temperature. The optimal temperature varied daily depending on pullet age, ranging between 39 °C and 27 °C.

**Table 1:** Locations and vaccination types used in the study.

Name	Location	Breed	Set-up date	Age at vaccination	Vaccination type	Pullets per location
Dataset 1	House 1 (Farm 1)	Lohmann Brown Classic	29-12-2022	3 weeks (week 3.1)	Live *Salmonella* enteritidis and *Salmonella* typhimurium at day 15 after set-up in drinking water (F0491A; Elanco)	30,000
Dataset 2	House 2 (Farm 1)	Lohmann LSL Classic, Lohmann LSL Lite, Lohmann Brown Classic	29-12-2022	3 weeks (week 3.2)	Live *Salmonella* enteritidis and *Salmonella* typhimurium at day 15 after set-up in drinking water (F0491A; Elanco)	75,000
Dataset 3	House 1 (Farm 1)	Lohmann Brown Classic	29-12-2022	12 weeks	Injection at day 82 after set-up with inactivated *Erysipelothrix rhusiopathiae* (H428A08 and H491A08; Intervet Nederland B.V), Avian infectious bronchitis virus, Newcastle disease virus, atadenovirus and avian rhinotracheitis virus (H856A12; Intervet Nederland B.V)	30,000
Dataset 4	House 3 (Farm 2)	Lohmann LSL Lite	11-11-2022	16 weeks	Live *Salmonella* enteritidis and *Salmonella* typhimurium (F0402A and F0491A; Elanco) at day 110 after set-up in drinking water	25,000

All pullets were vaccinated against a variety of diseases during the flock round as part of standard vaccination practices on commercial rearing farms. The vaccination of interest for House 1 was the *Salmonella* vaccination at wk 3 of age (Dataset 1) and the injections with a vaccine cocktail at wk 12 of age, consisting of *Erysipelothrix rhusiopathiae*, Avian infectious bronchitis virus, Newcastle disease virus, atadenovirus and avian rhinotracheitis virus (Dataset 3). In House 2, the *Salmonella* vaccination at wk 3 of age (Dataset 2) was of interest and in House 3 the *Salmonella* vaccination at wk 16 (Dataset 4). The *Salmonella* vaccination was administered through the drinking water, while the cocktail of bacterial and viral agents was delivered via 2 concurrent injections into the breast muscle. To allow for the latter vaccination, the pullets were again locked up in the aviary system a day beforehand. During the vaccination, the lights were completely turned off. Feed and water were withheld several hours before and during vaccination. Each rearing hen was picked up to receive the vaccination. For details on the flocks and vaccinations, see Table 1.

Because the study only involved vaccination procedures according to recognized animal husbandry, it was exempt from approval of animal use by the local ethical committee according to DIRECTIVE 2010/63/EU Article 1 5. (f).

### Sample collection

Manure was sampled daily from 2 days before vaccination to 4 days after vaccination for all houses. Fresh manure samples were collected daily on chick rearing paper (0.20 m^2^) that was placed on 8 fixed locations for 14-16 h, of which 6-10 h in the light period. The 8 locations were randomly spread over 1 or 2 of the 3 manure belts per house either at the back end of the belt or at both the front and the back end of the manure belt. The location selection ensured that hens were not disturbed during manure sample collection and was dependent on the specific design of the different houses. The sampling protocol guaranteed that pooled manure samples were exclusively collected from hens physically located within the same segregated subsection of the rearing hen house when they had unrestricted access to litter. The 8 collected samples were pooled together to 4 samples per day with fixed combinations, resulting in 24 (6 days x 4 pooled) manure samples per dataset. Each pooled manure sample weighed an average of 52 grams (ranging from 5 to 83 grams, depending on the amount of manure available on the chick rearing paper). Samples were stored in labeled zip-lock plastic bags. Samples analyzed within 2 h after collection were kept at room temperature, while those analyzed within 24 h were stored at 4 °C. House temperature and humidity were measured during the manure sampling days in at least 4 locations inside every house. Temperature and humidity during sample collection ranged between 15 °C - 33 °C and 45 % - 85 %, respectively.

### Analytical methods

*Volatile trapping.* In total, 96 manure samples were analyzed. Volatiles from the manure samples were trapped within 24 h after collection. Manure volatiles were concentrated into stainless steel sorbent tubes (Markes Tenax TA, USA). In short, a subsample of 1 g manure was taken after thoroughly mixing the pooled manure samples at 19-20 °C ambient temperature. The subsample was placed in a clean glass tube with a volume of 15 mL, which was connected to a mass flow controller (Bronkhorst FLOWSUITE v1.0.5.0, the Netherlands). The glass tube containing the subsample was flushed for 5 min with inert nitrogen with a flow of 50 mL min^-1^ to clear residue air out of the tube. After flushing, volatiles were sampled from the manure for 20 min with a nitrogen flow of 50 mL min^-1^ at 19-20 °C ambient temperature, and captured in the sorbent tubes. Sorbent tubes were immediately sealed with brass end caps. All sorbent tubes were analyzed within 2 months after volatile trapping.

*Gas chromatography-mass spectrometry system measurements.* Volatile analysis was carried out by a thermal desorption system (Markes International UltraTD / Unity2, USA) coupled with gas chromatography-mass spectrometry (**GC-MS**; Agilent GC 7890A and Agilent MS 5975, Agilent Technologies, USA). The Thermal Desorber was used to desorb the volatiles from the sorbent tubes. Flow path temperatures were held at 150 °C. First, tubes were heated to 320 °C for 15 min and continuously flushed for 15 min with helium (flow rate 60 mL min^-1^) to desorb volatiles. Volatiles were absorbed onto Tenax-coated traps at 25 °C with a flow of 60 mL min^-1^ and pre-purge flow on the trap is 60 mL min^-1^ for 1 min. Second, traps were heated to 300 °C with a maximum heating rate and a hold time of 5 min before injection on the gas chromatography system (**GC**) column. The split ratio was 1:23.4. The GC separated desorbed volatiles on a Restek 30 m x 320 µm x 0.25 µm Rtx-1 GC capillary column (Restek, USA), using a helium carrier gas (Messer Helium 5.0, Germany) at a constant pressure of 7.86 PSI. The GC oven temperature program was the following: 1) initial temp, 40 °C hold 7.3 min; 2) ramp 5.5 °C min^−1^ to 230 °C. The temperature of the mass spectrometry system (**MS**) was held at 230 °C for the MS source and 150 °C for the MS Quad.

*Data processing and analysis.* The raw GC-MS files (chemstation .D format) were converted into .abf files in Reifycs Analysis Base File Converter (ABF converter) and then processed with MS-Dial (V4.9.221218). In the data collection tab, the mz range was set to 0-450 Da, whereas the retention time (**RT**) range was limited to 0-66 min. For the Peak detection, a minimum peak height of 800 amplitude was selected and the ‘Accurate MS’ was checked as a high resolution GC-MS was used. For the smoothing of the peaks, the linear weighted moving average method was used with smoothing level of 3. Finally, the alignment, the ‘gap filling by compulsion’ was unchecked. Other settings were left on their original default. The data had to be batch corrected as the day of GC-MS measurement showed a batch effect. Here, the ComBat method was used, as it does not require any internal standards or quality control samples, via the MetaboAnalyst online platform (Johnson et al., 2007; Pang et al., 2024).

The resulting feature table (table with intensities of detected RT – mass spectrum MS combination for each of the samples) was sliced into smaller datasets for comparisons of specific samples. Feature tables were uploaded to MetaboAnalyst (V6.0; (Pang et al., 2024)) for data visualization. Here, feature tables were normalized by sum, log transformed and pareto scaled. First, Principal Component Analysis (**PCA**) was conducted to see any grouping of the samples based on factors, such as age, breed, etc. For the PCA plots, principle component (**PC**) 1 and 2 were plotted against each other, while for the control dataset, PC2 versus PC3 was additionally plotted. Then, Partial Least Squares Discriminant Analysis (**PLS-DA**) with 5-fold cross validation was applied using the factor ‘age’ for the control dataset or ‘time’ for the individual datasets. The PLS-DA was also used to get the Variables Importance In Projection (**VIP**) scores. Volatiles with high VIP scores (equal to or above 1.5) indicate differences in intensities in at least 1 of the factors. To assess the PLS-DA model, cross validation of the R2 (goodness of fit) and Q2 (goodness of prediction) performance measures were checked against the number of components. An R2 value equal to or above 0.7 and a Q2 value equal to or above 0.5 were considered good. If the values were below the thresholds, fold-change analysis of these datasets would be considered. For Dataset 4, samples before (days -2 and -1 pooled) and after vaccination (days 3 and 4 pooled) were portrayed in a volcano plot, as no overlap was shown in the PCA plot. Features with high VIP scores or significant levels in the fold-change analysis were selected for putative annotation, using a hybrid search (Cooper et al., 2019) with NIST spectral database (NIST MS Search 2.2, 2014) (Supplemental Table 1). The annotated features were used to make heatmaps with hierarchical clustering, using average distance done with the pheatmap package (1.0.12) in RStudio (2023.09.1; Build 494). Furthermore, to make the heatmaps comparable to each other, the data was scaled to have a range from -1 to 1. Venn diagrams were used to visualize the overlap between volatiles following the same trends and were generated by the webtool of Bioinformatics & Evolutionary Genomics of VIB/UGent (https://bioinformatics.psb.ugent.be/webtools/Venn/).

## Results

The study aimed to investigate the relationship between management-related stressors, induced by 2 types of vaccinations at 3 different ages, and the volatile profile of rearing hen manure on flock-level in a commercial setting.

Manure volatile profiles were identified and compared between different layer breeds, sample locations in the cage-free rearing house and ages of the pullets. The effect of a drinking water-based *Salmonella* vaccination, and an injection of a vaccine cocktail containing viruses and bacteria on manure, was studied by taking several manure samples before and after vaccination. Volatile compound and their peak intensities in the headspace above the samples were analyzed across several rearing houses. In total, 296 volatiles were found.

### Manure volatile profiles affected by age

As mentioned, the first step was to see whether or not other factors than vaccination would affect the manure volatile profiles. Therefore, samples before the vaccination event (day -2 and day -1) were used for PCA analysis (Supplemental Figure 1). The PCA explained 38 % (PC1 + PC2) of the total variation. The data points were labeled based on several factors: dataset, age, breed and sample location. For dataset, breed and sample location no clusters were found in the PCA plot and thus did not affect the manure volatile profiles before vaccination (Supplemental Figure 1 A, C and D). Age, on the contrary, showed a grouping across PC2 (Supplemental Figure 1 B). The samples of the datasets 1,3 and 4 are separated across PC1 and PC2, while samples of Dataset 2 are overlapping with Dataset 1 and Dataset 3. The separation of factors age and dataset became clearer when looking at PC2 and PC3 (Supplemental Figure 2). However, 2 clusters across PC1 were found, but could not be explained by any of the factors.

**Fig. 1:**
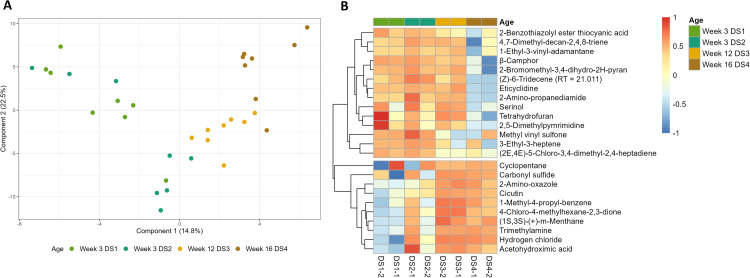
Manure volatiles of controls (day -2 and day -1) of each dataset. Coloring based on age of the rearing hen flock. 3-week old flocks are indicated by light green for Dataset 1 and dark green for Dataset 2, whereas the light brown 12-weeks old and dark brown 16-weeks-old. A) Partial Least Squares Discriminant Analysis (PLS-DA) and B) Heatmap of identified volatiles with a Variables Importance In Projection (VIP) score above 1.5. Volatiles with high peak intensities were shown in red, and blue represents volatiles with low peak intensity.

As age was the only factor that showed clustering in the PCA plot, the supervised version PLS-DA was employed (Fig. 1 A). Here, the same clustering based on age (young vs old) can be seen. With the PLS-DA, volatiles with high variable in projection (VIP, ≥1.5) score were selected. The selected volatiles were visualized in a heatmap (Fig. 1 B, Supplemental Figure 3). The heatmap shows 2 clusters: 1) high peak intensity at wk 3 and low peak intensity at wk 12/16 and 2) low peak intensity at wk 3 and high peak intensity at wk 12/16. Cluster 1 consisted of 14 volatiles, whereas cluster 2 consisted of 10 volatiles.

### Changes in volatilome due to vaccination

**Fig. 2:**
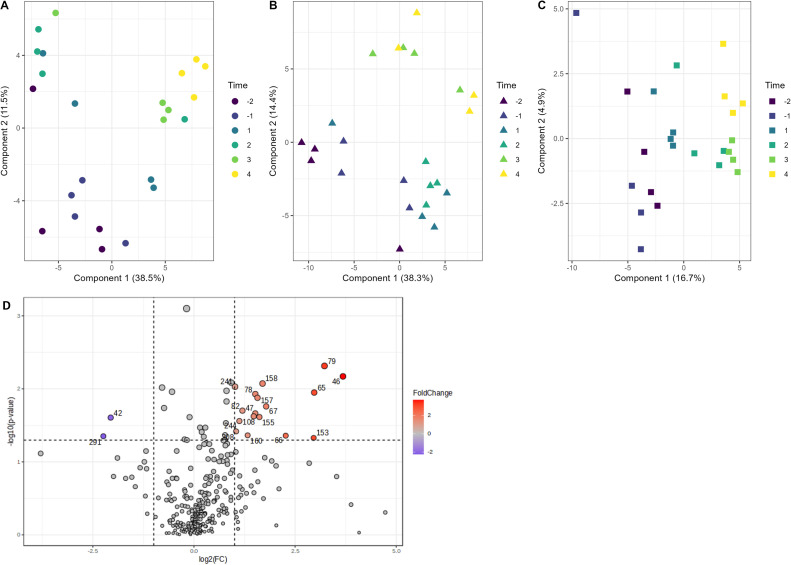
Volatiles in the manure of rearing hens 2 days before and 4 days after vaccination of different flock. Partial Least Squares Discriminant Analysis (PLS-DA) for A) Dataset 1, B) Dataset 2 and C) Dataset 3. Color gradient from dark blue to yellow indicates the time progression from 2 days before the vaccination event to 4 days afterwards. D) Volcano plot for Dataset 4 for the comparison of the control samples (day -2 and -1) against vaccinated samples (day 3 and day 4). Blue indicates lower in the vaccinated samples compared to control, whereas red indicated higher. 42 = Pivalonitrile, 46 = Furan (Retention time (RT) = 1.662), 47 = Oxazole, 65 = Triisopropylborane, 66 = Butanoic acid, 67 = Spiro[2,4]hepta-4,6-diene, 78 = Unknown (RT = 4.542), 79 = 2,5-Dimethylpymrimidine, 82 = Unknown (RT = 5.076), 108 = 1,3-Dimethyl-3-n-butyldiaziridine, 153 = (Z)-6-Tridecene (RT = 21.011), 155 = 4,7-Dimethyl-decan-2,4,8-triene, 157 = 2-Bromomethyl-3,4-dihydro-2H-pyran, 158 = Beta-camphor, 160 = Eticyclidine, 208 = 3-Ethyl-3-heptene, 241 = Alpha-chloropinacolone, 244 = 1-Ethyl-3-vinyl-adamantane, and 291 = Unknown (RT = 48.173).

The second step was to detect changes in manure volatile profile due to vaccination. Manure was sampled for 2 days before vaccination and 4 days after vaccination. A PCA was used to compare volatiles over time for each of the datasets, with 4 replicates per sampling day (Supplemental Figure 4). The PCAs showed no straightforward time-trends, except for Dataset 3 (Supplemental Figure 4C). Here, a clear separation over PC1 between before vaccination, including 1 day after vaccination and day 2 until 4 after vaccination, was seen. The other datasets showed some separation between before and after vaccination. However, there was an overlap between these groupings. For Dataset 1 and Dataset 2, where the pullets received a *Salmonella* vaccination at 3 weeks of age, cross-validated PLS-DA plots showed a clear separation of samples across time along components 1 and 2 (Fig. 2A and Fig. 2B), with 50 % of total variation explained for Dataset 1 and 52.7 % for Dataset 2. A similar pattern was seen for Dataset 3, where the pullets received an injection at 12 weeks of age, although the PLS-DA explained 21.6 % of total variation (Fig. 2C), which was less than for Dataset 1 and Dataset 2. Cross-validation of the PLS-DA model for Dataset 4, where the pullets received a *Salmonella* vaccination at the age of 16 weeks, revealed an insufficient predictive ability (Q2≤ 0.2), meaning that there was no clear distinction in volatiles between samples in time. Therefore, a volcano plot was used to analyze the orientation, intensity, and statistical significance of the changes in volatile peak intensities between manure samples collected before vaccination (day -2 and day -1) versus after vaccination (day 3 and day 4) (Fig. 2D). These days were selected because little to no overlap was observed in the PCA plot (Supplemental Figure 4D). The exact fold changes of volatiles are represented in Supplemental Table 2.

**Fig. 3:**
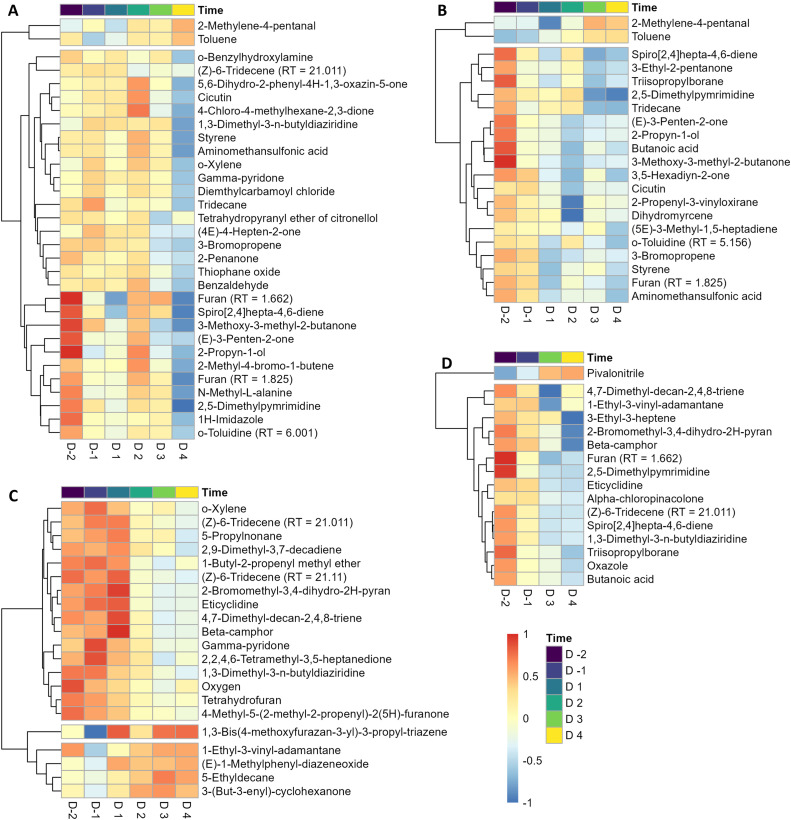
Changes in average manure volatile intensity over time for each dataset. Heatmaps have hierarchical clustered volatiles which could be annotated. Volatiles for Dataset 1-3 had VIP score ≥ 1.5, whereas for dataset volatiles had p-value ≤ 0.05 and fold change ≥ 1. Columns of the heatmaps are individual days with negative numbers indicting days before the vaccination event whereas positives number the days after vaccination. A) Heatmap for Dataset 1. B) Heatmap for Dataset 2. C) Heatmap for Dataset 3. D) Heatmap for Dataset 4. Volatiles with high peak intensities were shown in red, and blue represents volatiles with low peak intensity.

The PLS-DA models and Volcano plot identified differential volatiles between sampling days during vaccination moments for each dataset. The normalized and scaled peak intensities of differential volatiles were visualized in heat maps across time per dataset to show the effect of vaccination (Fig. 3, Supplemental Figures 5-8). Dataset 1 (Fig. 3A) showed 2 different clusters. The first cluster has 2 volatiles, whose peak intensities increased over time. The second cluster consisted of 29 volatiles with decreased intensities over time, either slowly going down over time (18 volatiles) or fast going down over time (11 volatiles). Dataset 2 (Fig. 3B) showed similar clusters as Dataset 1, although in another order, with 13 volatiles going down fast over time and 6 volatiles going down slowly over time, and 2 volatiles with increased peak intensity over time. Dataset 3 (Fig. 3C) showed 3 separate clusters. In the first cluster, 16 volatiles had a decreased peak intensity over time. In the second and third cluster, respectively 1 volatile showed an irregular pattern and 4 had an increased intensity over time. In Dataset 4 (Fig. 3D), 2 clusters could be identified, with 1 volatile increasing and 15 decreasing over time.

### Stress markers

Volatiles that showed differential peak intensities across vaccination time per dataset were compared between datasets to see whether or not vaccination similarly affected manure volatile profiles across rearing hen houses, ages, and types of vaccination. Venn diagrams give an overview of volatiles shared between datasets that went up or down over time during the vaccination event (Supplemental Figure 9). Table 2 shows the largest overlap of volatiles that increased after vaccination between Dataset 1 and Dataset 2, where the pullets from 2 separate flocks received a Salmonella vaccination at wk 3 of age. 11 volatiles were reduced in both Dataset 1 and Dataset 2 after vaccination, whereas 2 were increased in both datasets.

**Table 2:** Overlap of volatile peak intensity changes found between the 4 datasets (DS) in response to the vaccination event.

Overlap between datasets^1^	Trend over time^2^	Volatiles
DS 1 and DS2	Going up over time	Toluene, 2-Methylene-4-pentanal
DS1 and DS 2	Going down over time	3-Bromopropene, Tridecane, Styrene, Furan (RT = 1.825), 2-Propyn-1-ol, Aminomethansulfonic acid, Cicutin, 3-Methoxy-3-methyl-2-butanone, (E)-3-Penten-2-one
DS1 and DS 3	Going down over time	1,3-Dimethyl-3-n-butyldiaziridine, Gamma-pyridone
DS1, DS3 and DS4	Going down over time	1,3-Dimethyl-3-n-butyldiaziridine, (Z)-6-Tridecene (RT = 21.011)
DS1 and DS 4	Going down over time	Furan (RT = 1.662)
DS1, DS 2 and DS 4	Going down over time	2,5-Dimethylpymrimidine, Spiro[2,4]hepta-4,6-diene
DS 2 and DS 4	Going down over time	Butanoic acid, Triisopropylborane
DS3 and DS4	Going down over time	Beta-camphor 2-Bromomethyl-3,4-dihydro-2H-pyran 4,7-Dimethyl-decan-2,4,8-triene Eticyclidine

1 DS 1 = week 3 of age, DS 2 = week 3 of age, DS 3 = week 12 of age, DS 4 = week 16 of age.

2 Volatiles were split into 2 groups either going up or down over time during the vaccination event. Combinations not mentioned in the table, do not show overlap.

Between Dataset 3 and Dataset 4, after the injection vaccination at the age of 12 weeks, and the Salmonella vaccination at the age of 16 weeks, respectively, 6 volatiles showed a decrease in peak intensity (Supplemental Figure 9). No volatile showed a consistent increase or decrease in peak intensity across all datasets.

## Discussion

Intestinal dysbiosis is known to have a key influence on the general health, welfare and performance of laying hens. Although fecal droppings are a direct indicator of gut health, manual scoring of color, consistency, and pathogen-load is time-consuming, subjective, and labor-intensive, especially in large-scale non-cage houses. The present study aimed to investigate the indicative value of volatile organic compounds for stress experienced by pullets based on fecal droppings from a commercial poultry house. The study aimed to determine the extent to which vaccination events influence the manure volatile compound profile, considering the potential for age-dependent and vaccine-type-dependent effects on the strength and direction of the stress response. The results showed that several volatiles from rearing hen manure are affected by vaccination, with overlapping volatile response to *Salmonella* in the young pullets, and few overlapping responses with *Salmonella* and multi-pathogen injections in the older pullets. In contrast, no volatiles showed a similar response to vaccination when comparing all datasets across houses, suggesting that the age of the pullets and type of vaccination are important factors when analyzing the response of the pullets to vaccination as a proxy for a stress event.

### Effect of age on volatile profile

The manure volatile compound profile of the pullets before vaccination appeared to differ between the pullets of 3 weeks of age and 12 to 16 weeks of age, with beta-camphor, (Z)-6-Tridecene, and serinol peak intensities being elevated in the young pullets and trimethylamine (TMA) and carbonyl sulfide being more dominant in the older pullets. The variation in volatile peak intensities between ages might be attributed to the natural development of the gut microbiome in laying hens over time, thus affecting volatile volumes released from manure (Ducatelle et al., 2018; Shirasu and Touhara, 2011). As hens mature, the microbiome undergoes substantial changes, particularly in the cecum, which is a key site for microbial fermentation and volatile organic compound production. For example, the relative abundance of *Proteobacteria* in the cecum tends to decrease as hens age (Kers et al., 2018; Sun et al., 2021), whereas the abundance of *Bacteroidetes* generally increases (Sun et al., 2021). Additionally, the abundance of *Firmicutes,* characterized by potentially beneficial bacteria, may either increase or decrease with age, depending on various factors, such as diet, microbiota disturbance in early life, and environment (Kers et al., 2018; Simon et al., 2016; Sun et al., 2021).

These microbiome shifts likely drive the changes in volatile profiles observed in the feces of the pullets at different ages in this study. As hens age, the increasing prevalence of *Firmicutes* may contribute to the increased production of TMA, given that bacteria in this phylum are known to metabolize choline and carnitine into TMA (Rath et al., 2017). TMA is a volatile found in animal intestines for break-down of nitrogen-containing compounds (https://hmdb.ca/metabolites/HMDB0000906). Conversely, the decrease in *Proteobacteria* might be related to the reduced availability of beta-camphor as an energy source. Beta-camphor is produced by soil micro-organisms and linked to plant – micro-organism communication (Russo et al., 2022). In this study, it remains uncertain whether or not beta-camphor was reduced over time due to the presence of camphor-degrading *Proteobacteria* in the intestinal tract of the pullets or in the poultry house's drinking lines (Kanehisa laboratories, 2023; Maes et al., 2020).

Although it has been argued that layer breed affects microbiota composition (Kers et al., 2018), we did not find an effect of layer breed on volatiles in fecal droppings. Instead, the manure volatiles in our study might have been affected by feed composition. The diet of young hens significantly differs from that of older hens, and dietary changes are among the primary factors that can modify the balance and mutualistic relationship between gut microbiota and the host (Turnbaugh et al., 2009). This suggests that age-related changes in diet, rather than genetic differences among breeds, play a crucial role in shaping the gut microbiota and its metabolic outputs.

### Similarities and differences in vaccination response across different ages

Our study shows that young pullets might be differently affected by *Salmonella*-induced stress than older pullets. The slightly larger variance explained in the PLS-DA suggests that *Salmonella* vaccination-induced stress responses have a greater impact on volatile profiles at 3 weeks of age compared to 16 weeks of age. This age effect could be due to differences in immune system development and microbiome development. The young pullets exhibited the most consistent changes in volatile peak intensities after *Salmonella* vaccination despite being housed in separate rearing houses, with compounds such as aminomethanesulfonic acid decreasing after *Salmonella* vaccination. Aminomethanesulfonic acid, a sulfur-containing compound with an intermediate structure between glycine and taurine (Ivica et al., 2022), has shown relevance in modulating immune responses. After hatch, young hens have a more underdeveloped adaptive immune system than older hens, and rely to a greater extent on innate immune responses, where microbes trigger host immune cells non-specifically to produce chemokines, cytokines and host defense peptides within minutes or hours after exposure (Alkie et al., 2019; Meijerink et al., 2021). It is known that aminomethanesulfonic acid can bind to receptors that reduce the production of inflammatory cytokines, like TNFα, suggesting a potential role in managing inflammation and stress responses following vaccination (Ishizaki-Koizumi et al., 2004). The presence of this compound in the volatile profiles of the young pullets highlights the modulation of innate immune processes engaged in response to vaccination. The reduction in aminomethanesulfonic acid, especially steeply at day 4 after vaccination, may suggest a protective dampening of the inflammatory response against live *Salmonella*. The absence of a similar response in aminomethanesulfonic acid peak intensity in the older pullets could be due to the relative stability of their adaptive immune system, which takes several days or weeks to respond to vaccination, beyond the duration of our manure sample window (Meijerink et al., 2021; Withanage et al., 2003). Moreover, the *Salmonella* vaccination dose is consistent across all ages and likely not adjusted for bird body weight. This may result in a reduced stress impact on older pullets compared to younger pullets. In general, the smaller effect of *Salmonella* vaccination on volatile profiles at 16 weeks of age compared to 3 weeks of age might be due to the relative stability of the gut microbiome against pathogens, such as *Salmonella,* in older chickens (Gast, 2008) compared to younger chickens. Environmental factors, such as exposure to litter areas and variable temperature and humidity conditions from wk 4 of age onwards, may have introduced additional diversity and complexity in volatile manure profiles in the older pullets and diminished the differentiating effect of vaccination (Dunlop et al., 2016; Kers et al., 2018).

Despite the apparent greater impact of *Salmonella* vaccination on manure volatiles in hens at 3 weeks of age than 16 weeks of age, several volatiles showed a similar decrease in peak intensity after vaccination, such as 2,5-Dimethylpymrimidine, Spiro[2,4]hepta-4,6-diene and furans, even across different houses. Furan peak intensity decreased after *Salmonella* vaccination at both ages. Furans are a product of bacterial metabolism, where furanose is produced from dietary fructose, particularly under anaerobic conditions. Probert et al. (2004) showed a positive relation between furans and *Clostridium difficile* infection in abnormal human fecal samples. The change in furan peak intensity in this study might indicate a stabilization phase of the microbiota composition and activity. Previous studies showed that oral administration of live *Salmonella* strains to day-old chicks can effectively protect chicks against infection with related *Salmonella* organisms within hours by an intestinal colonization–inhibition effect, which is most certainly the result of microbial physiological processes (Methner et al., 2011). After vaccination with live *Salmonella* strains, colonization of the caeca occurs very quickly, and cecal levels of *Salmonella* gradually start to decrease after a few days (Cawthraw et al., 2023). Vaccination could have cross-inhibited colonization of *Clostridium Difficile* after vaccination, leading to reduced furan in manure samples both in young hens with immature microbiomes and older hens with more stable microbiomes.

The study revealed an apparent decrease in (Z)-6-Tridecene levels in 3 out of 4 vaccination events. This volatile decreased after Salmonella vaccination in only 1 flock of young pullets, whereas in older pullets, it decreased in both flocks following the injection at wk 12 and the Salmonella vaccination at wk 16. (Z)-6-Tridecene is an unsaturated aliphatic hydrocarbon, which is typically associated as byproducts of membrane lipid peroxidation (https://hmdb.ca/metabolites/HMDB0061827). This compound has been identified at higher levels in inflammatory conditions, such as chorioamnionitis in humans, although not significantly different from healthy individuals (Ophelders et al., 2021). The observed decrease post-vaccination suggests that the immune response may modulate oxidative stress and lipid peroxidation, leading to reduced production of (Z)-6-Tridecene. Moreover, (Z)-6-Tridecene can be degraded by anaerobic bacteria, which might be influenced by changes in the gut microbiota composition after vaccination (Grossi et al., 2008). This decrease may also reflect improved immune regulation and reduced physiological stress, as unsaturated aliphatic hydrocarbons are upregulated in health deficits, like cancer melanoma (Abaffy et al., 2010). Additionally, the young pullets in this study showed higher levels of (Z)-6-Tridecene before vaccination than the older pullets, which might relate to increased inflammatory stress in young chicks. These findings highlight the potential role of (Z)-6-Tridecene as a biomarker for metabolic and immune changes in response to vaccination in poultry.

### Effect of vaccination effectiveness, method and antigen load on vaccination response

Despite similarities in response to *Salmonella* vaccination at wk 3 of age between the 2 rearing flocks, a few differences have been observed. In House 2, slightly more variance was explained in the PLS-DA due to *Salmonella* vaccination at 3 weeks of age. The higher variance explained in House 2 may be attributed to increased sample homogeneity and a more consistent environment compared to House 1. In House 2, samples were taken exclusively from the middle manure collection belt, corresponding to pullets kept on 1 level of the rearing system, whereas samples from House 1 were taken from both the middle and lower manure collection belts. Additionally, Cawthraw et al. (2023) showed that only 53 % of birds vaccinated at 5 days of age had detectable levels of *Salmonella* in cecal and liver samples 2 days after vaccination, indicating that vaccination effectiveness and individual sensitivity to vaccines can vary between pullets and potentially influence the volatile peak intensity changes observed in this study.

Besides vaccination effectiveness, also the method and infectious load of vaccine administration might affect the perceived stress load thus the effect of vaccination on volatile profile of pullets. The vaccination event at wk 12 of age combined several stressful situations, such as locking up the pullets in the dark for several hours and handling the pullets for injection that induces pain. Even though administered vires were inactivated, total viral load was high. This could explain the improved separation of samples across time for this vaccination event, as opposed to the vaccination response at 16 weeks of age, where the vaccine was provided in the drinking water and manure samples did not form a gradient in time based on volatile profile. The amount of perceived stress might thus affect the magnitude and direction of volatile changes in rearing hen manure.

### The potential for disease detection and optimization

Most studies have examined poultry manure volatiles in the context of malodorous environmental pollution, but these volatiles may also have potential as markers of intestinal health, as observed in human research (Ducatelle et al., 2018). In general, we did not find a similarity in vaccination-induced differential volatiles across all 4 datasets. The absence of any overlapping vaccination-induced differential volatiles could be due to external factors around sample collection that affect manure volatile profiles independent of vaccination stress, such as variability in temperature, humidity, diet, or other farm management practices. Despite the large spatial variation in manure characteristics, such as pH, total solids and ammonia concentrations that have been found in laying hen houses (Li and Ni, 2021), sample location within the rearing house did not seem to affect the manure volatile profile before vaccination. This finding is promising when regarding the implementation of sensors inside the poultry house to detect changes in health on flock-level.

The absence of specific vaccination-induced biomarkers for hen health status could alternatively imply that vaccinations are no consistent, effective, straightforward proxies for stress events, and it remains unknown to what extent the vaccinations in our study led to reduced intestinal health. Moreover, manure volatiles might not be a straightforward measure of stress, as volatile organic compounds are the result of microbiota metabolism, the host metabolism and immune function, and host-microbiota interaction. Instead, it would be insightful to additionally measure changes in inflammation-induced or dysbiosis-induced host proteins and microbiota composition as a result of vaccination or any other management-related stressor (Ducatelle et al., 2018). To further investigate the relationship between volatiles and hen health, volatile sampling during other (stressful) events, such as poultry red mite infestation—an issue particularly prevalent in cage-free laying hens—could be valuable, as severe infestations are associated with notable behavioral changes and production losses (Kilpinen et al., 2005; Sleeckx et al., 2019).

Our candidate volatiles for stress indication have not been observed in similar studies examining stress-induced manure or air volatiles in poultry or other livestock species. Only styrene, which was decreased in peak intensity after *Salmonella* vaccination at wk 3 of age, has also been found in broiler farm air, both without chicks present and with chicks present across all ages of the broiler flock (Dunlop, 2011). We initially expected to detect short-chain fatty acids and other fatty acids in the manure samples, with potential changes in their peak intensities linked to the age and hen health status (Mozuriene et al., 2024). It is known that stressors, such as exposure to high temperatures, can significantly increase the levels of acetate and total volatile fatty acids in manure samples from laying hens, indicating heightened bacterial fermentation (Gomez-Osorio et al., 2021; Kim et al., 2020). However, fatty acids are highly volatile, and their detection is complicated. To improve detection, the volatile extraction method could be adapted to Ultra-High-Performance Liquid Chromatography UHPLC with mass spectrometry analysis based on freeze-dried feces, as headspace solid-phase microextraction of chicken feces has been shown to lack reproducibility in other studies (Beauclercq et al., 2019; Bernard et al., 2024). Another opportunity for optimization would be narrowing the manure sample window from 14-16 h to a shorter period to limit variability in feces freshness, which can be influenced by factors such as temperature (Berkhout et al., 2016). Variability in freshness, along with differences in manure consistency, dry matter content, and pH, affects volatile profiles (Mozuriene et al., 2024). Processing refinement is essential in volatile analysis from organic materials. In many studies, several volatiles are identified and reported that are of non-natural origin. For example, benzaldehyde is likely formed by the degradation of the Tenax tube, and reactive atmospheric gases like hydrogen chloride can lead to the loss of meaningful volatiles (Dettmer and Engewald, 2002). Caution is necessary when identifying volatiles and interpreting their potential role in chemical communication to avoid validating findings based on studies that mistakenly implicate molecules known to be contaminants (Charpentier et al., 2012).

Promising, reliable biomarkers for monitoring intestinal health will likely be stable molecules present in feces and litter that can be easily quantified. Combining these biomarkers with other techniques, such as machine learning-based vision technologies on abnormal feces detection (He et al., 2024) could enhance the accuracy and reliability of health monitoring systems. Odor-based monitoring approaches are often underrepresented in reviews on precision livestock farming (Goossens et al., 2022; Li et al., 2020; Morrone et al., 2022), and studies often focus on disease monitoring in broiler farm air. Volatiles from manure could provide a more consistent profile of poultry health over time than volatiles in farm air, with greater proximity to the source when aiming to detect deviations in intestinal health. Continued research in these areas could contribute to innovative tools for improving health monitoring and management practices in poultry farming, such as e-noses to detect specific gastro-intestinal diseases. These could aid poultry farmers in objective health deviation detection and ultimately the prevention of clinical disease outbreaks on poultry farms.

## Conclusion

Manure volatile profiles of pullets change in response to vaccination as a proxy for stress, but the response depends on hen age and vaccination method, effectiveness and pathogen load. Although no vaccination-induced differential volatiles were found across all 4 datasets, the reduced peak intensities of (Z)-6-Tridecene after vaccination in 3 out of 4 flocks suggest a potential for detecting deviations in pullet manure based on volatile profile. Future research could transition from exploratory studies to more extensive investigations that account for variables such as diet, housing conditions, environmental factors, and management practices that affect hen manure. These studies should aim to identify significant differences in specific manure volatile compounds before and after relevant stressors in pullets, providing deeper insights into their potential as reliable markers for poultry stress or health monitoring.

## Declaration of competing interest

The authors declare that they have no known competing financial interests or personal relationships that could have appeared to influence the work reported in this paper.

## Appendix Supplementary materials

Image, application 1
