# Supplementary material for: Manure odor profiling for flock-level monitoring on commercial layer pullet farms: Vaccination events as a model stressor
Source: Poult Sci. 2024 Dec 16;104(2):104681. doi: 10.1016/j.psj.2024.104681 (PMC11729669; doi:10.1016/j.psj.2024.104681)
Supplement: Supplementary file 1 [file mmc1.docx]

SUPPLEMENTARY DATA

**Supplemental Table 1. Annotated Volatiles of all datasets.** Includes unknown volatiles.

| Volatile | Retention time (min) |
| --- | --- |
| Oxygen | 0.954 |
| Unknown (RT = 1.105) | 1.105 |
| Serinol | 1.113 |
| (4E)-4-Hepten-2-one | 1.122 |
| 3-Bromopropene | 1.124 |
| Unknown (RT = 1.147) | 1.147 |
| Unknown (RT = 1.165) | 1.165 |
| (5E)-3-Methyl-1,5-heptadiene | 1.172 |
| Trimethylamine | 1.268 |
| 2-Methylene-4-pentanal | 1.309 |
| Unknown (RT = 1.314) | 1.314 |
| Cyclopentane | 1.367 |
| Unknown (RT = 1.368) | 1.368 |
| Hydrogen chloride | 1.381 |
| Acetohydroximic acid | 1.399 |
| 3-Ethyl-2-pentanone | 1.403 |
| Thiophane oxide | 1.416 |
| Thioacetic acid | 1.421 |
| Unknown (RT = 1.448) | 1.448 |
| unknown (Rt = 1.472) | 1.472 |
| 1H-Imidazole | 1.481 |
| Unknown (RT = 1.488) | 1.488 |
| Pivalonitrile | 1.543 |
| 2-Pentanone | 1.57 |
| Tetrahydrofuran | 1.641 |
| Furan (RT = 1.662) | 1.662 |
| Oxazole | 1.667 |
| Unknown (RT= 1.721) | 1.721 |
| 2-Amino-oxazole | 1.728 |
| Cicutin | 1.747 |
| Furan (RT = 1.825) | 1.825 |
| N-Methyl-L-alanine | 1.835 |
| Carbonyl sulfide | 2.082 |
| Unknown (RT = 2.317) | 2.317 |
| Unknown (RT= 2.345) | 2.345 |
| Triisopropylborane | 2.374 |
| Butanoic acid | 2.618 |
| Spiro[2,4]hepta-4,6-diene | 2.711 |
| Toluene | 2.738 |
| Unknown (RT = 3.116) | 3.116 |
| 3-Methoxy-3-methyl-2-butanone | 3.266 |
| (E)-3-Penten-2-one | 3.314 |
| 2-Propyn-1-ol | 3.32 |
| Unknown (RT = 4.542) | 4.542 |
| 2,5-Dimethylpymrimidine | 4.845 |
| Gamma-pyridone | 4.846 |
| Unknown (RT = 4.925) | 4.925 |
| Unknown (RT = 5.076) | 5.076 |
| o-Toluidine (RT = 5.156) | 5.156 |
| Styrene | 5.158 |
| Aminomethansulfonic acid | 5.225 |
| Dimethylcarbamoyl chloride | 5.82 |
| 3,5-Hexadiyn-2-one | 5.825 |
| o-Benzylhydroxylamine | 5.983 |
| Unknown (RT = 5.986) | 5.986 |
| o-Toluidine (RT = 6.001) | 6.001 |
| Unknown (RT = 6.85) | 6.85 |
| Benzaldehyde | 8.555 |
| 2-Oxo-3-phenyl-1,3-oxazetidine | 8.583 |
| 2-Amino-propanediamide | 8.597 |
| Methyl vinyl sulfone | 9.422 |
| Unknown (RT = 10.166) | 10.166 |
| N-Aminomorpholine | 10.813 |
| 1,3-Dimethyl-3-n-butyldiaziridine | 11.941 |
| o-Xylene | 12.529 |
| 5,5-Dimethyl-1-vinylbicyclo[2.1.1]hexane | 12.529 |
| Unknown (RT = 12.613) | 12.613 |
| 2-Propenyl-3-vinyloxirane | 12.796 |
| 3-Phenyl-3-buten-2-one | 13.193 |
| 1-Methyl-4-propyl-benzene | 13.316 |
| 1,4-diethyl-benzene | 13.469 |
| Unknown (RT = 14.133) | 14.133 |
| 4-Chloro-4-methylhexane-2,3-dione | 14.209 |
| 1-tert-Butyl-2-methylbenzene | 15.507 |
| 2E)-3-Methyl-2-nonene | 15.582 |
| Di-tert-butyl ketone | 15.586 |
| 5,6-Dihydro-2-phenyl-4H-1,3-oxazin-5-one | 16.442 |
| α-Oxobenzeneacetonitrile | 17.059 |
| (1S,3S)-(+)-m-Menthane | 17.331 |
| unknown (RT = 17.611) | 17.611 |
| N-(4-Iodo-phenyl)-3,4-dimethyl-benzamide | 17.974 |
| Silicon-based compound | 17.977 |
| 4-(1H-pyrrol-1-yl)-Phenol | 18.657 |
| 1-Chloro-2-methylbutane | 19.099 |
| (2Z)-5,5-Dimethyl-2-hexene | 19.965 |
| Dihydromyrcene | 20.463 |
| 2-Methyl-4-bromo-1-butene | 20.62 |
| 5-Propylnonane | 20.746 |
| 4-Methyl-5-(2-methyl-2-propenyl)-2(5H)-furanone | 20.959 |
| 1-Butyl-2-propenyl methyl ether | 20.963 |
| Unknown (RT = 21) | 21 |
| (Z)-6-Tridecene (RT = 21.011) | 21.011 |
| Unknown (RT = 21.041) | 21.041 |
| 4,7-Dimethyl-decan-2,4,8-triene | 21.044 |
| 2,9-Dimethyl-3,7-decadiene | 21.049 |
| 2-Bromomethyl-3,4-dihydro-2H-pyran | 21.062 |
| Beta-camphor | 21.11 |
| (Z)-6-Tridecene (RT = 21.11) | 21.11 |
| Eticyclidine | 21.117 |
| Unknown (RT = 21.399) | 21.399 |
| (E)-1-Methylphenyl-diazeneoxide | 21.405 |
| Tridecane | 21.418 |
| Unknown (RT = 21.425) | 21.425 |
| Unknown (RT = 21.446) | 21.446 |
| 2-Naphthalenecarbonitrile | 21.457 |
| Unknown (RT = 21.594) | 21.594 |
| 1,3-Bis(4-methoxyfurazan-3-yl)-3-propyl-triazene | 21.608 |
| 2,2,4,6-Tetramethyl-3,5-heptanedione | 21.623 |
| 3-Ethyl-3-heptene | 21.9 |
| Unknown (RT = 22.481) | 22.481 |
| (2E,4E)-5-Chloro-3,4-dimethyl-2,4-heptadiene | 22.54 |
| Unknown (RT = 22.69) | 22.69 |
| Alpha-chloropinacolone | 23.01 |
| 1-Ethyl-3-vinyl-adamantane | 23.075 |
| 2-Ethylbutyl bromide | 23.184 |
| Unknown (RT = 27.958) | 27.958 |
| Unknown (RT = 28.481) | 28.481 |
| Unknown (RT = 28.492) | 28.492 |
| 5-Ethyldecane | 28.5 |
| 3-(But-3-enyl)-cyclohexanone | 28.561 |
| Unknown (RT = 34.72) | 34.72 |
| Tetrahydropyranyl ether of citronellol | 37.993 |
| Unknown (RT = 38.018) | 38.018 |
| 2-Benzothiazolyl ester thiocyanic acid | 42.269 |
| Unknown (RT = 48.173) | 48.173 |
| Unknown (RT = 48.479) | 48.479 |
| Unknown (RT = 58.376) | 58.376 |

**Supplemental Table 2. Fold changes (FC) and p-value belonging to the volcano plot of Dataset 4.** Includes unknown volatiles.

| **Volatile** | **ID** | **Retention time (min)** | **Log2(FC)** | **-LOG10(p-value)** |
| --- | --- | --- | --- | --- |
| Pivalonitrile | 42 | 1.543 | -2.0591 | 1.6059 |
| Furan (RT = 1.662) | 46 | 1.662 | 3.6836 | 2.1704 |
| Oxazole | 47 | 1.667 | 1.5154 | 1.6648 |
| Triisopropylborane | 65 | 2.345 | 2.9727 | 1.9496 |
| Butanoic acid | 66 | 2.618 | 2.268 | 1.359 |
| Spiro[2,4]hepta-4,6-diene | 67 | 2.711 | 1.785 | 1.7596 |
| Unknown (RT = 4.542) | 78 | 4.542 | 1.5167 | 1.9284 |
| 2,5-Dimethylpymrimidine | 79 | 4.845 | 3.2265 | 2.3137 |
| Unknown (RT = 5.076) | 82 | 5.076 | 1.1982 | 1.7012 |
| 1,3-Dimethyl-3-n-butyldiaziridine | 108 | 11.941 | 1.4762 | 1.6238 |
| (Z)-6-Tridecene (RT = 21.011) | 153 | 21.011 | 2.9593 | 1.3279 |
| 4,7-Dimethyl-decan-2,4,8-triene | 155 | 21.044 | 1.6185 | 1.6138 |
| 2-Bromomethyl-3,4-dihydro-2H-pyran | 157 | 21.062 | 1.5711 | 1.8773 |
| Beta-camphor | 158 | 21.11 | 1.6954 | 2.0726 |
| Eticyclidine | 160 | 21.117 | 1.3275 | 1.3633 |
| 3-Ethyl-3-heptene | 208 | 21.9 | 1.0421 | 1.4174 |
| Alpha-chloropinacolone | 241 | 23.01 | 1.0162 | 2.0289 |
| 1-Ethyl-3-vinyl-adamantane | 244 | 23.075 | 1.1224 | 1.559 |
| Unknown (RT = 48.173) | 291 | 18.173 | -2.239 | 1.3511 |


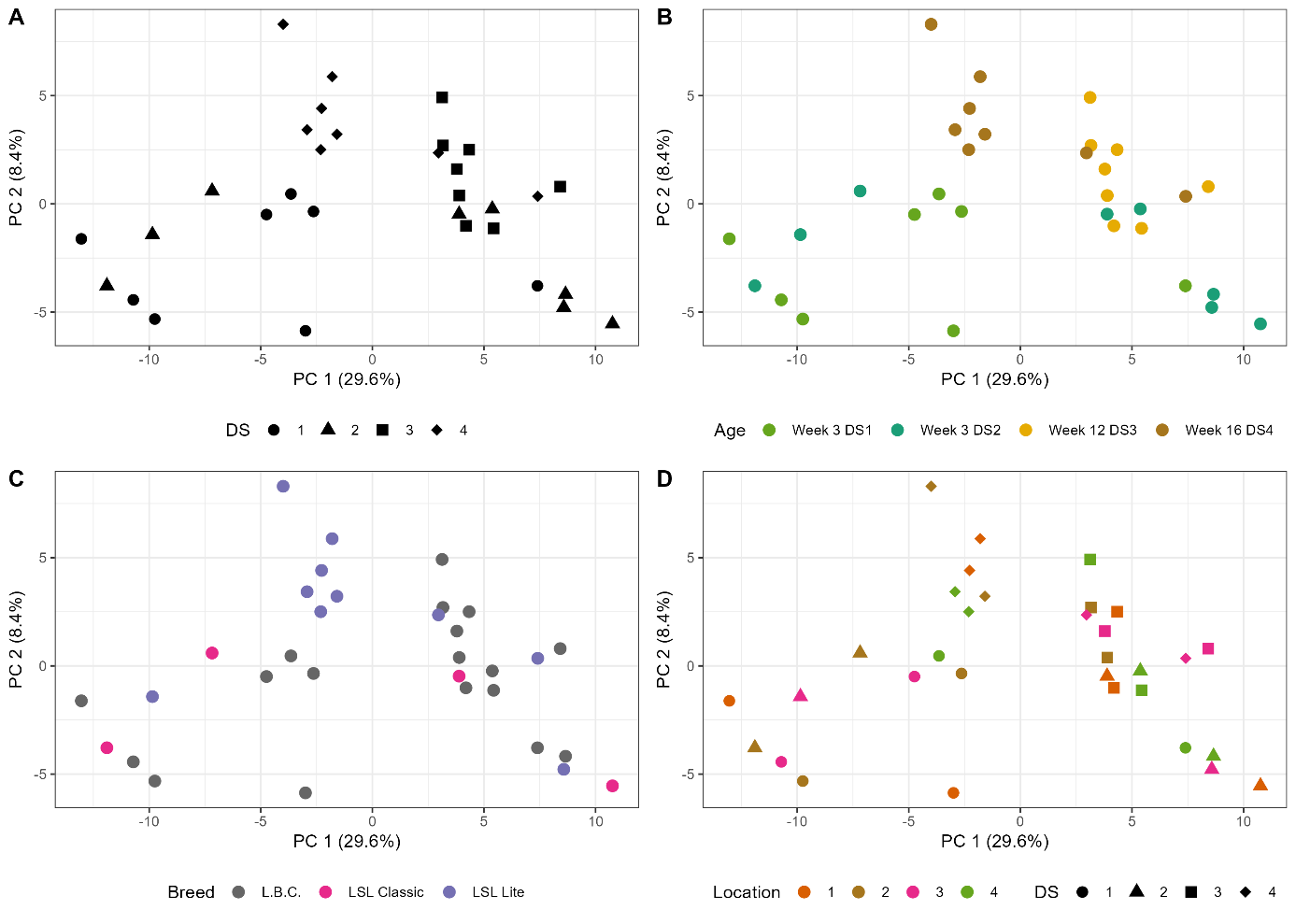
**Supplemental Figure 1: Principal component analysis (PCA, PC1 vs PC2) plots for the control samples which were taken 2 days before vaccination event.** A) PCA plot based on dataset (DS). Round shape = Dataset 1, triangle = Dataset 2, square = Dataset 3, and diamond = Dataset 4. B) PCA plot based on age. Green colors indicate 3-week old flock, light brown = 12-weeks old flock, and dark-brown = 16-weeks old flock. C) PCA plot based on breed. Gray = Lohmann Brown Classic (L.B.C), pink = LSL Classic, and purple = LSL Lite. D) PCA plot based on sample location. Orange = location 1, brown = location 2, pink = location 3, and green = location 4. Shapes as in A).


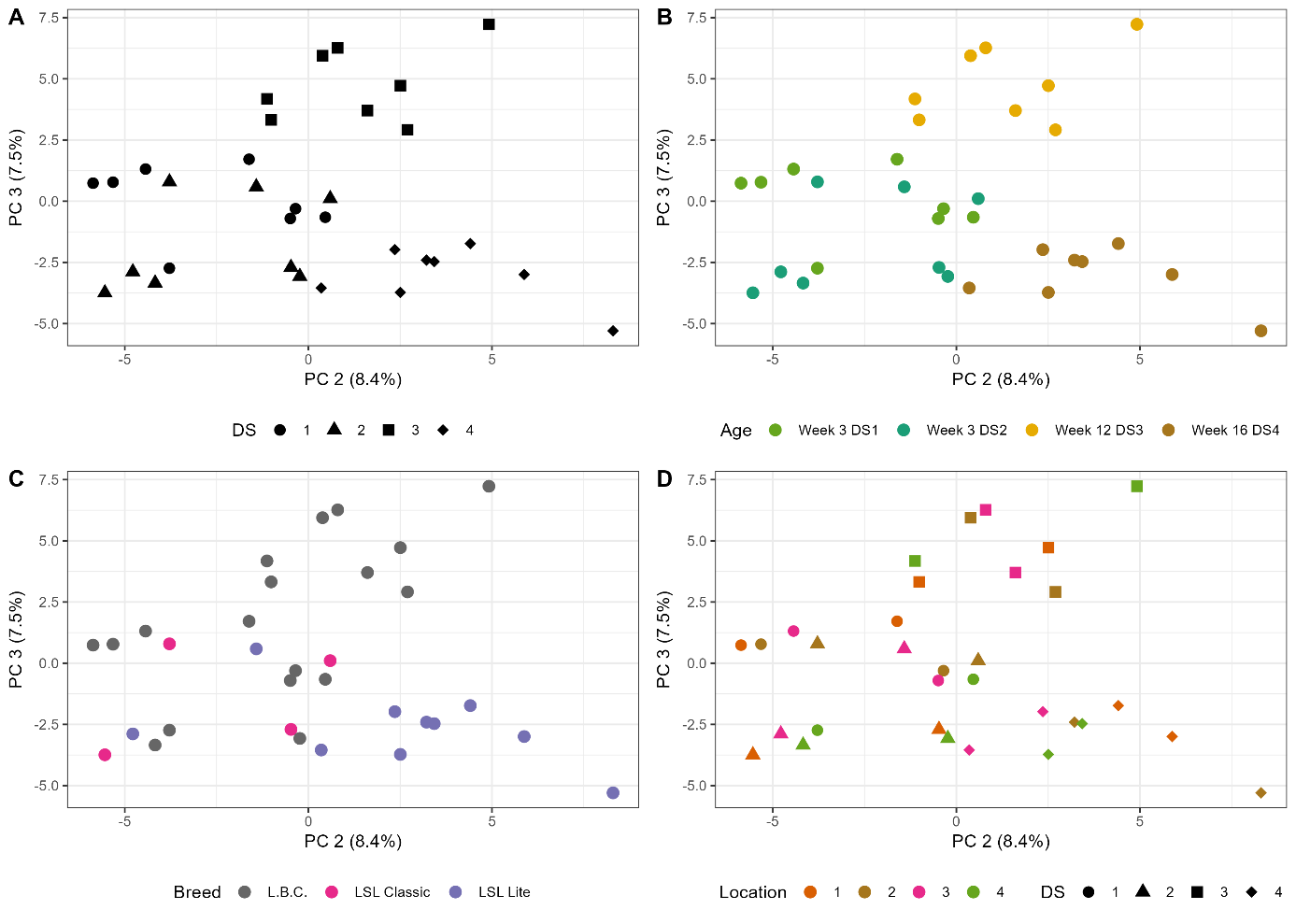
**Supplemental Figure 2: Principal component analysis (PCA, PC2 vs PC3) plots for the control samples which were taken 2 days before vaccination event.** A) PCA plot based on dataset (DS). Round shape = Dataset 1, triangle = Dataset 2, square = Dataset 3, and diamond = Dataset 4. B) PCA plot based on age. Green colors indicate 3-week old flock, light brown = 12-weeks old flock, and dark-brown = 16-weeks old flock. C) PCA plot based on breed. Gray = Lohmann Brown Classic (L.B.C), pink = LSL Classic, and purple = LSL Lite. D) PCA plot based on sample location. Orange = location 1, brown = location 2, pink = location 3, and green = location 4. Shapes as in A).


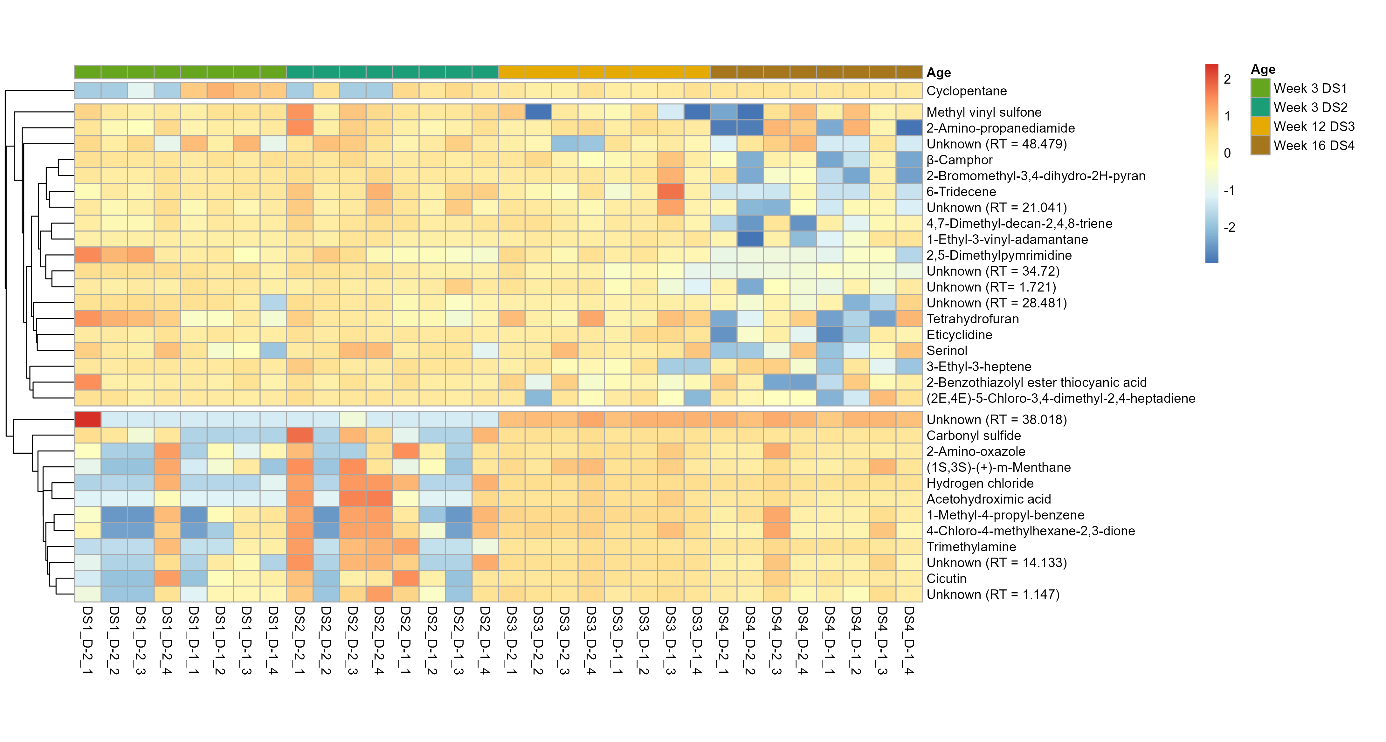
**Supplemental Figure 3: Extension of heatmap of control samples.** Volatile intensities of individual replicates shown (N = 4 per day per dataset (DS)). Additionally, unknown volatiles with a variable in projection (VIP) score ≥ 1.5 shown. Age displayed in top row of heatmap. Green colors indicate 2-week old flock, light brown = 12-weeks old flock, and dark-brown = 16-weeks old flock.


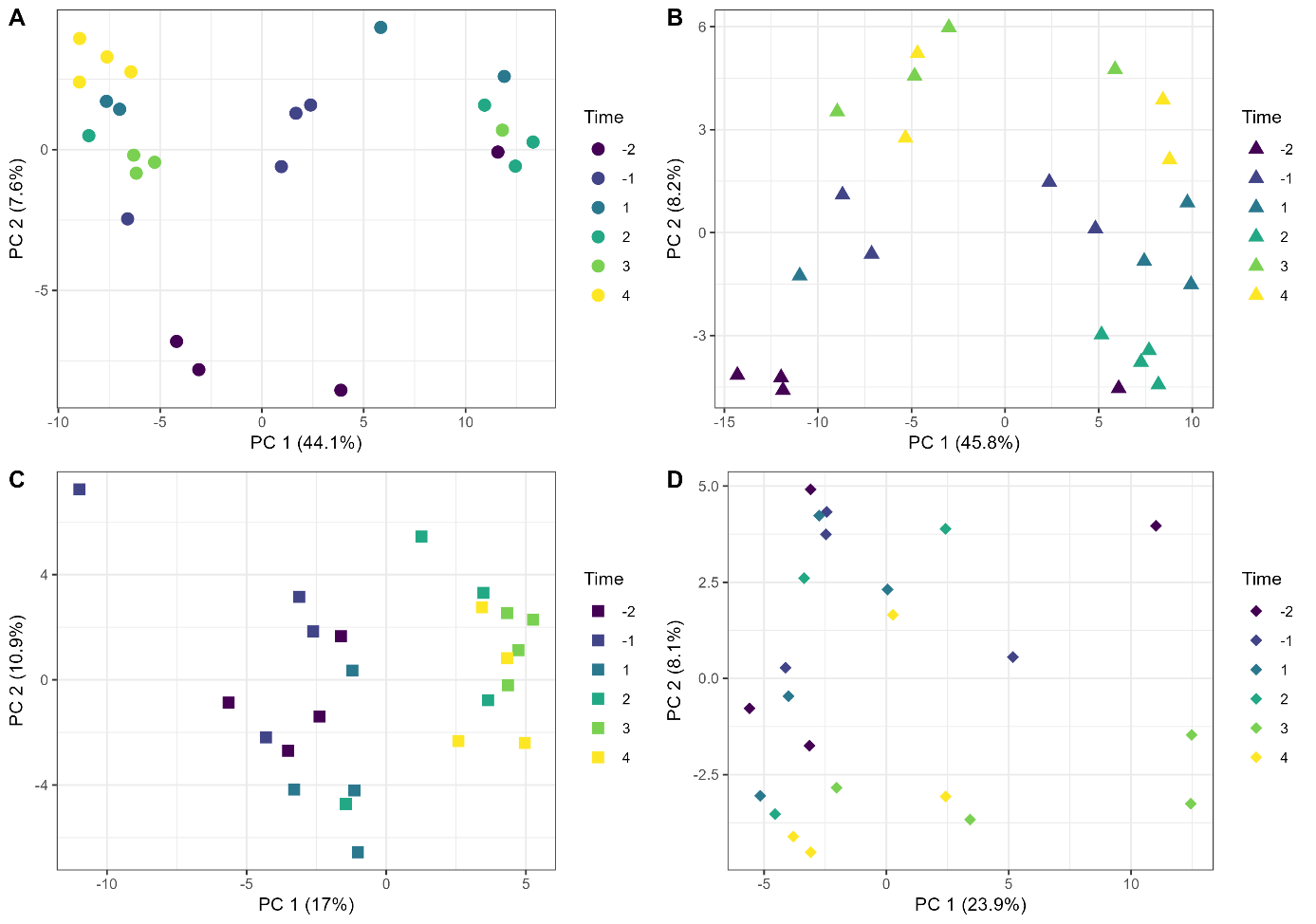


**Supplemental Figure 4: Principle component analysis (PCA, PC1 vs PC2) plots for each dataset.** Coloring from dark blue to yellow based on time which started 2 days before the vaccination and ended 4 days after. A) PCA for Dataset 1. B) PCA for Dataset 2. C) PCA for Dataset 3. D) PCA for Dataset 4.


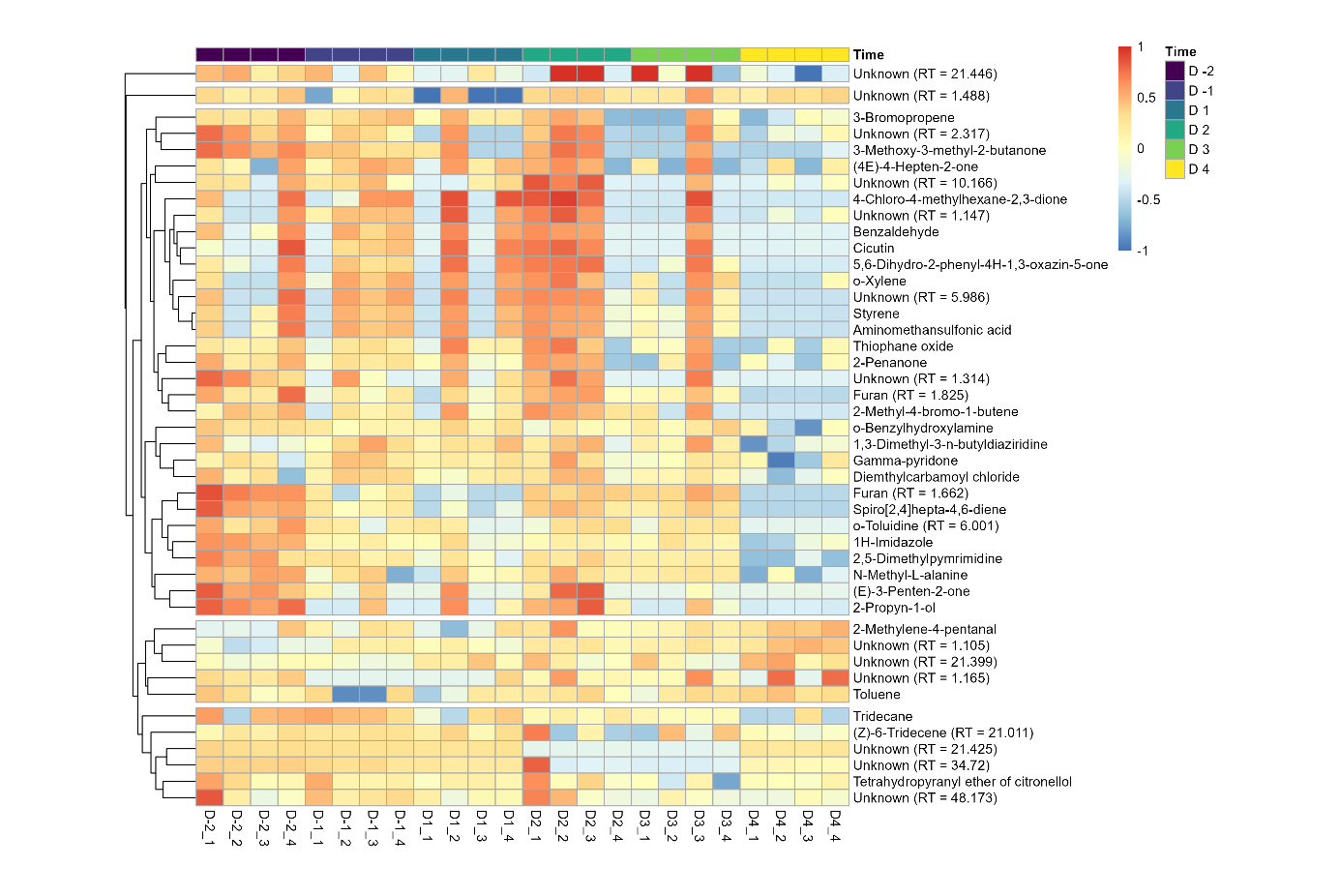
**Supplemental Figure 5: Extended heatmap of Dataset 1.** Volatile intensities of individual replicates shown (N = 4 per day) for Dataset 1. Additionally, unknown volatiles with a variable in projection (VIP) score ≥ 1.5 shown. Time displayed in top row of heatmap.


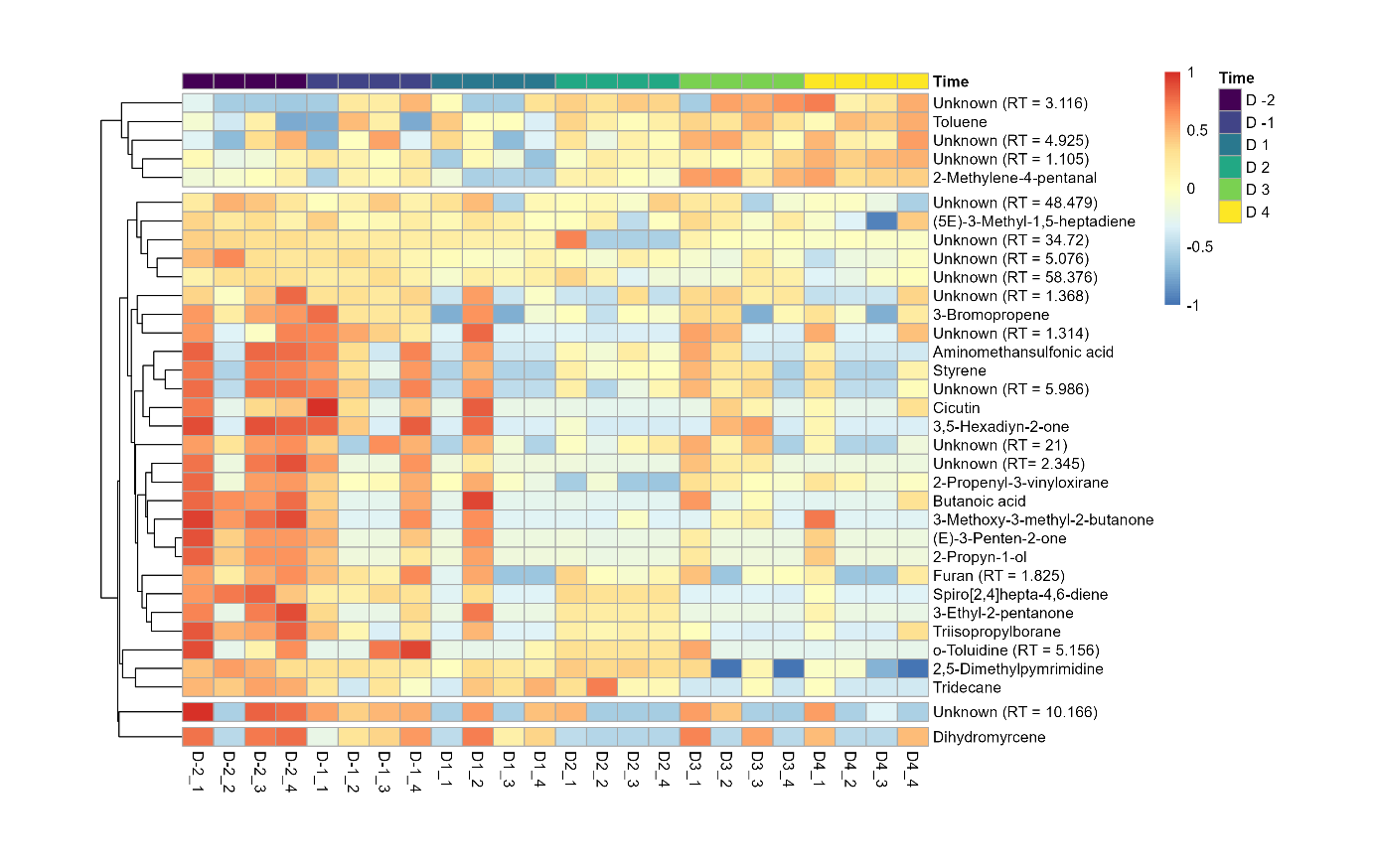
**Supplemental Figure 6: Extended heatmap of Dataset 2.** Volatile intensities of individual replicates shown (N = 4 per day) for Dataset 2. Additionally, unknown volatiles with a variable in projection (VIP) score ≥ 1.5 shown. Time displayed in top row of heatmap.


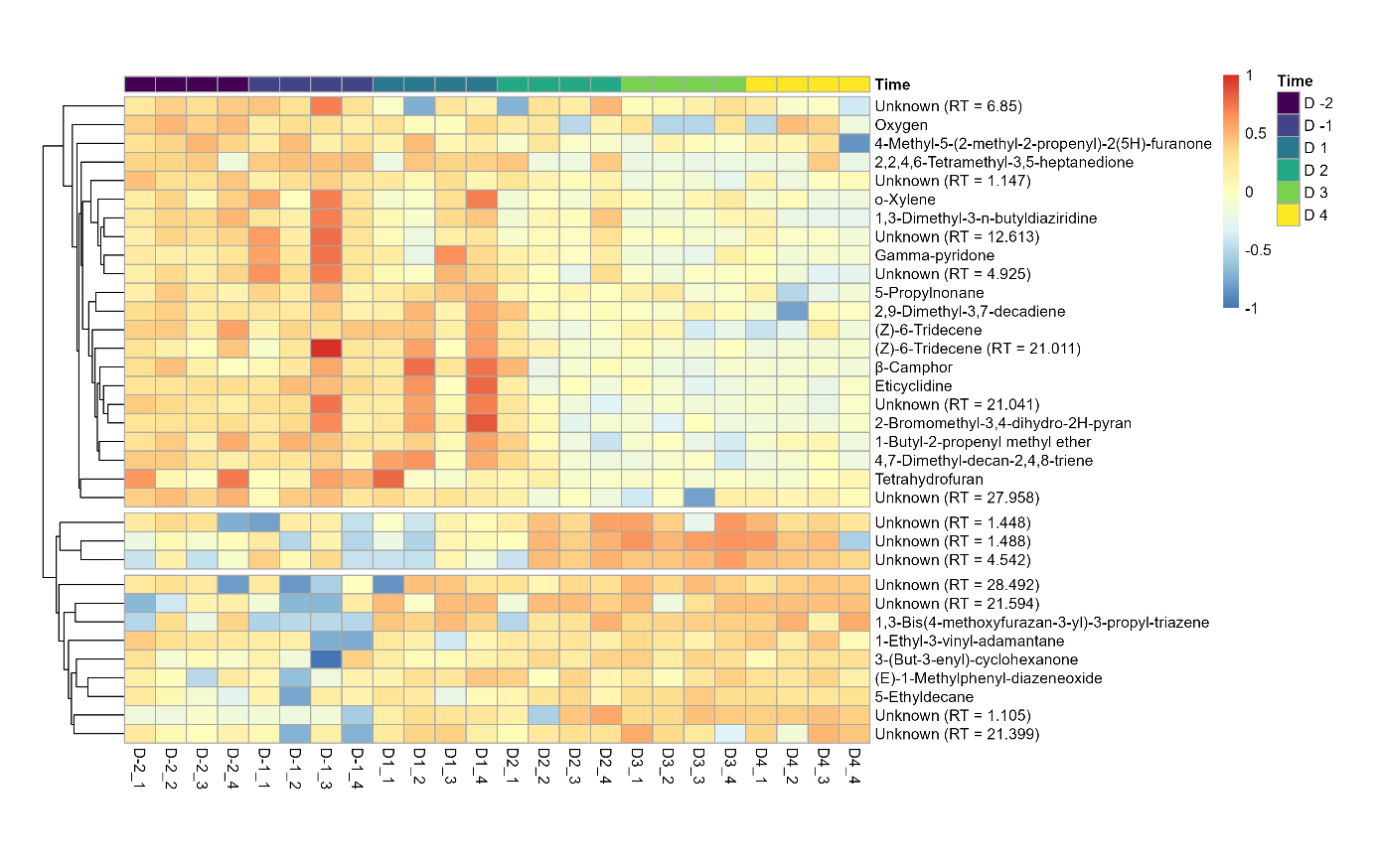
**Supplemental Figure 7: Extended heatmap of Dataset 3**. Volatile intensities of individual replicates shown (N = 4 per day) for Dataset 3. Additionally, unknown volatiles with a variable in projection (VIP) score ≥ 1.5 shown. Time displayed in top row of heatmap.


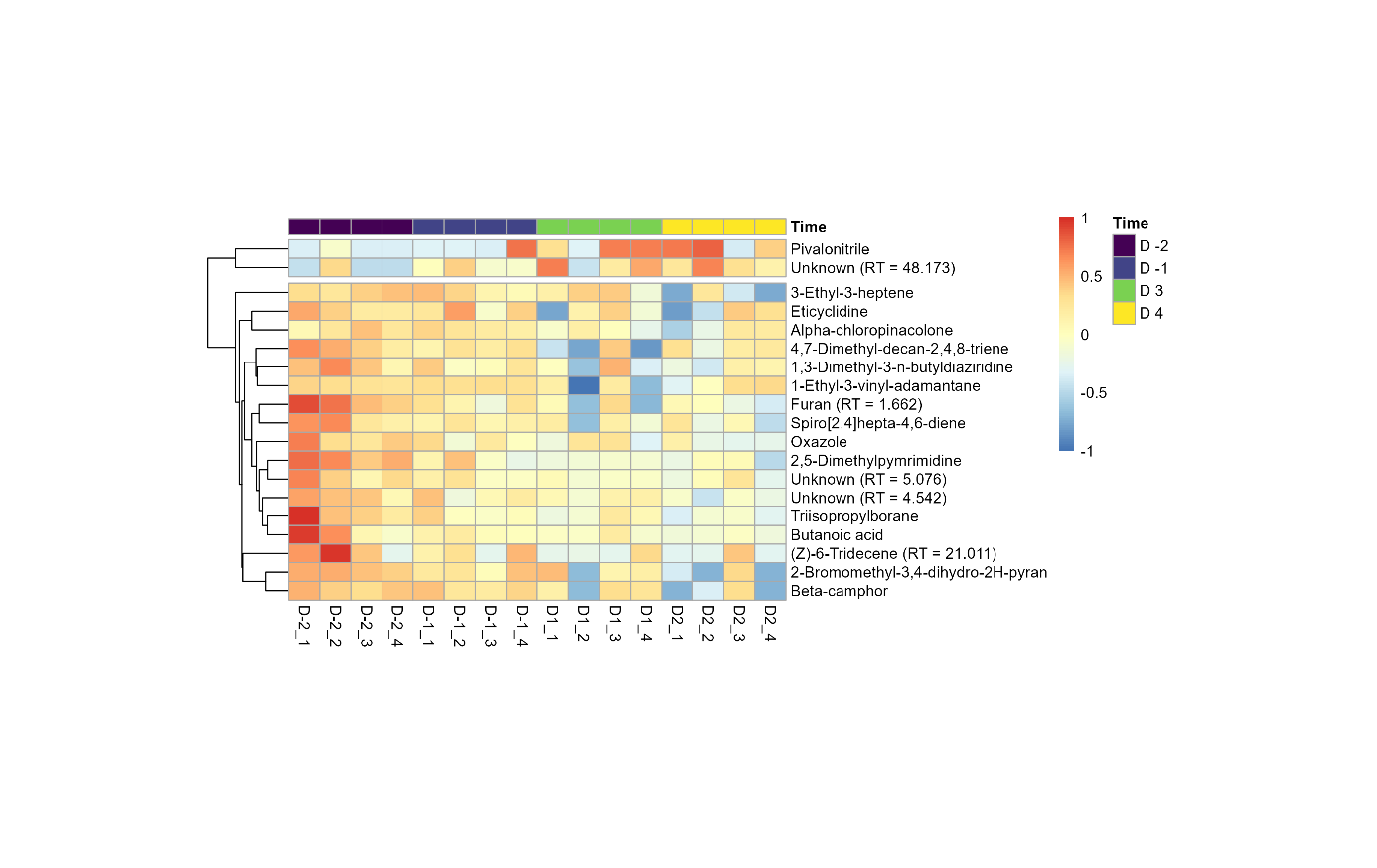


**Supplemental Figure 8: Extended heatmap of Dataset 4.** Volatile intensities of individual replicates shown (N = 4 per day) for Dataset 4. Additionally, unknown volatiles with a variable in projection (VIP) score ≥ 1.5 shown. Time displayed in top row of heatmap.


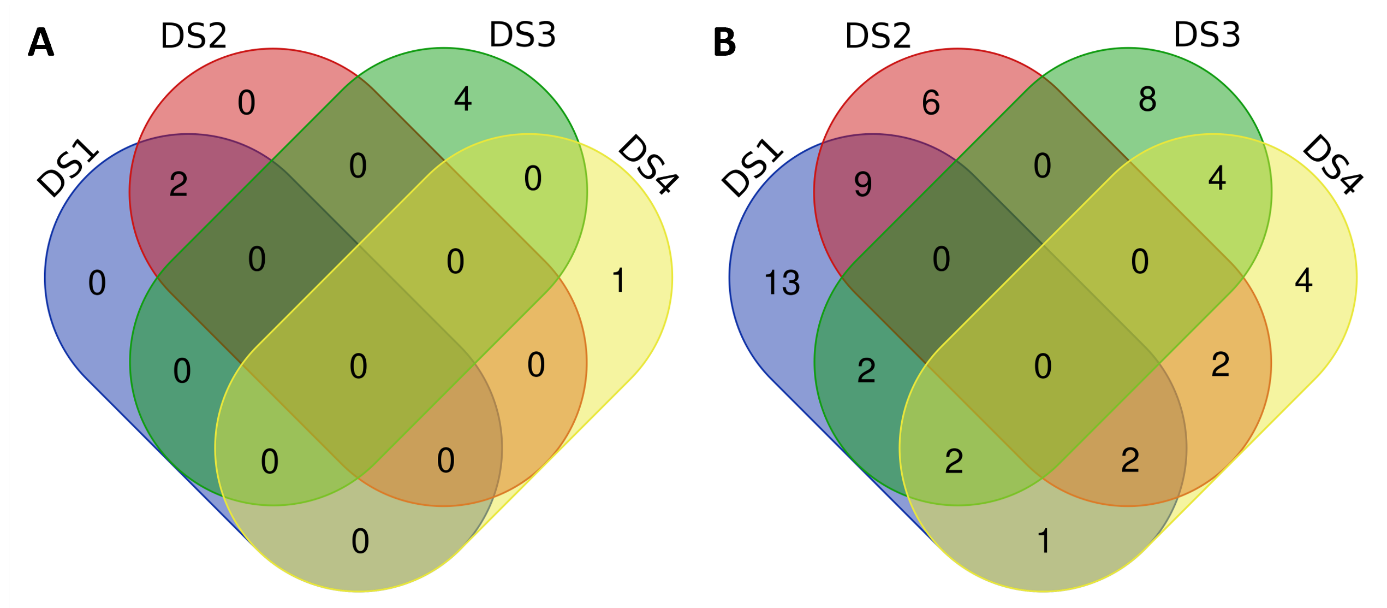


**Supplemental Figure 9: Venn diagrams of volatiles shared between datasets.** A) Overlapping volatiles that go up over time across all 4 datasets. B) Overlapping volatiles that go down over time across all 4 datasets. All diagrams exclude unknowns.
